# Hyperthermic Intraperitoneal Chemotherapy and Cytoreductive Surgery in Ovarian Cancer: An Umbrella Review of Meta-Analyses

**DOI:** 10.3389/fonc.2022.809773

**Published:** 2022-05-09

**Authors:** Amine Souadka, Hajar Essangri, Mohammed Anass Majbar, Amine Benkabbou, Saber Boutayeb, Benoit You, Olivier Glehen, Raouf Mohsine, Naoual Bakrin

**Affiliations:** ^1^ Surgical Oncology Department, National Institute of Oncology, University Mohammed V in Rabat, Rabat, Morocco; ^2^ Medical Oncology Department, National Institute of Oncology, University Mohammed V in Rabat, Rabat, Morocco; ^3^ Département d’oncologie médicale Centre Hospitalier Lyon-Sud, Hospices Civils de Lyon, Lyon, France; ^4^ Département de Chirurgie Digestive et Endocrinienne, Centre Hospitalier Lyon-Sud, Hospices Civils de Lyon, Lyon, France

**Keywords:** umbrella review, epithelial ovarian cancer, HIPEC, meta-analysis, peritoneal carcinomatosis (PC), cytoreductive surgery and HIPEC

## Abstract

**Background:**

The utility of heated intraperitoneal chemotherapy (HIPEC) in the management of epithelial ovarian cancer (EOC) has been assessed in several randomised clinical trials and meta-analyses, and it is still a subject of controversy. Therefore, we performed an umbrella review of existing meta-analyses to summarise the outcomes of HIPEC and cytoreductive surgery (CRS) association in ovarian cancer.

**Methods:**

We examined the MEDLINE, Cochrane Library, Scopus, Prospero, Web of Science and Science Direct from inception to May 30, 2020, for meta-analyses of randomised controlled trials and observational studies. Analyses of overall survival, disease free survival and progression survival were performed separately for primary and recurrent ovarian cancers.

**Results:**

We identified 6 meta-analyses investigating the association of HIPEC with CRS in the management of ovarian cancer. Three year overall survival was significantly improved by the association of CRS and HIPEC for primary (HR: 0.66, 95%CI:0.56-0.78) and recurrent ovarian cancers (HR:0.50, 95%CI:0.38-0.64). This benefit was also demonstrated on disease-free survival for primary (HR: 0.54, 95%CI:0.48-0.61) and recurrent ovarian cancer (HR: 0.60, 95%CI:0.46-0.78). The pooled hazard ratios confirmed the advantage of HIPEC and CRS association with respect to CRS alone on progression free survival for primary and recurrent ovarian cancer respectively with HR: 0.50, 95%CI: 0.43-0.58 and HR: 0.59, 95%CI: 0.41-0.85.

**Conclusion:**

While waiting for the results of the current prospective studies, the present umbrella study suggests that HIPEC performed at the end of CRS may be a complementary effective asset for ovarian cancer patient management.

## 1 Introduction

Epithelial ovarian cancer (EOC) is the most common cause of gynaecological cancer death worldwide, with a late onset diagnosis, high death-to-incidence rate and poor overall prognosis (1). While 70% of cases are detected in advanced stages, late stage presentations of the disease are associated with a 5-year relative overall survival rate of 29%, as opposed to 92% for early-stage disease (2, 3). Unlike other malignancies, the dissemination of ovarian cancer cells has a particular pattern which selectively invades the mesothelium of the peritoneal surface, spreading within the peritoneal cavity in a highly aggressive and rapidly growing manner, up to the encasement of reproductive organs and viscera (4). Consequently, the standard treatment consists of cytoreductive surgery (CRS), associated with systemic platinum-based chemotherapy (5–7).

CRS is an aggressive locoregional treatment involving the resection of the disseminated intra-abdominal disease (7, 8). This includes, hysterectomy, bilateral salpingo-oophorectomy, omentectomy as well as additional procedures such as peritonectomies (9), bowel resections, diaphragm peritonectomy with or without segmental full-thickness diaphragm resection, splenectomy with or without distal pancreatectomy, segmental liver resection, cholecystectomy, partial stomach resection, and partial bladder/ureteral resection (10). Postoperative residual disease was shown to be a major prognostic factor of overall survival (11–13), hence the necessity of surgery to be complete. In fact, surgery used to be qualified as “optimal” in reference to a residual tumour of less than 1 cm, but this is not the goal anymore. Currently, surgery is performed when expected to be complete with the aim of removing all macroscopic tumours, which can be challenging in cases of invasive carcinomatosis. In this context, the peritoneal cancer index (PCI) assessment at the time of surgical exploration is a valuable indicator which enables the estimation of the extent of carcinomatosis, the probability of complete cytoreduction and overall the oncological outcome (14, 15).

Besides its indications in the management of rare peritoneal cancers, the use of heated intraperitoneal chemotherapy (HIPEC) in association to CRS has been the subject of controversy. In fact, the OVHIPEC study was the first extensive phase III Randomised Clinical Trial to assess HIPEC benefit in the first line management of ovarian cancer (16). These results were subject to criticism (17), and although they led to guidelines change in some countries such as France, they did not have a similar impact on a more international level (18–20). At present, 17 ongoing clinical trials are examining the impact of HIPEC and CRS association in the management of primary or recurrent ovarian cancer (21). Whilst the results from these studies are pending, the synthesis of all available and robust data up to date is crucial. To assimilate the vast amount of research available on the effect of HIPEC and CRS in ovarian cancer, we performed an umbrella review of existing meta-analyses on this association to look at the impact on overall, disease free and progression free survival as well as morbidity and quality of life in primary and recurrent ovarian cancer.

## 2 Materials and Methods

Umbrella reviews can be referred to as overviews of reviews, reviews of reviews, a summary of systematic reviews and consist of the overall examination of the body of information on a specific subject/intervention in order to highlight similarities or contradictions in the results (22, 23). In view of the lack of widely accepted guidelines for umbrella reviews’ carry out, we followed the Cochrane Collaboration guidelines in conducting and reporting the results of this review (24). A protocol was priorly designed in accordance with the reporting guidance provided in the Preferred Reporting Items for Systematic Reviews and Meta-Analyses Protocols (PRISMA-P) statement, then registered within the international Prospective Register of Systematic Reviews (PROSPERO) database for systematic reviews and meta-analyses (CRD42020171008) (25).

### 2.1 Search Strategy

Two researchers (AS and HE) independently searched the MEDLINE, Cochrane Library, Scopus, Prospero, Web of Science and Science Direct from inception until May 30, 2020, to identify peer-reviewed meta-analyses of observational studies and randomised controlled trials. We also searched the references listed in eligible articles. No language restrictions were applied. Detailed search strategy is provided in **Appendix 1** and key words and MESH were defined as follows:

[Hyperthermic intraperitoneal chemotherapy’ OR ‘HIPEC’ OR ‘intraperitoneal’ OR ‘Intraperitoneal Chemotherapy, Hyperthermic’]
AND
[‘ovarian’ OR ‘ovary’ OR ‘Ovarian Neoplasm’ OR ‘Ovary Neoplasms’ OR ‘Neoplasm, Ovary’ OR ‘Ovarian Cancer OR Cancer’ OR ‘Cancer of the Ovary’]
AND
[“Cytoreduction Surgical Procedures”]
AND
[‘Meta-Analysis as Topic’ OR ‘Meta-Analysis ‘ [Publication Type].

### 2.2 Eligibility Criteria and Data Extraction

Review articles fulfilling the following criteria included (1): Meta-analyses of randomised controlled trials and observational prospective cohort studies (2) addressing ovarian cancer (3) examining the outcome of HIPEC in association with CRS (4) assessing overall survival and progression free or disease free survival.

Studies were excluded if they were primary studies, included the evaluation of HIPEC in other malignancies without separate analysis for ovarian cancer, and those for which it is not possible to retrieve the full article.

### 2.3 Data Collection and Analysis

The reviewed articles meeting the inclusion criteria were imported by each reviewer separately using a Zotero ^©^ software (version 5.0.80 for macOS), which is an open-source and free research tool for reference management (26). The duplicates were removed, and then the titles and abstracts of all articles were screened independently prior to full article review and final selection. A manual search of references cited in the selected articles was also performed to identify additional studies. Disagreements were resolved through consensus. When a consensus could not be reached a third investigator made the final decision. A list of excluded studies is provided.

### 2.4 Data Extraction and Management

Data extraction was performed independently by two investigators (AS and HE) and in case of discrepancies a third investigator was involved. A pre-established data extraction form was used to collect the following Information: (1) general information: title, author, journal name, year of publication (2) study characteristics: country, period of publication, number of original studies, type of intervention, comparator and type of outcomes. (3) outcome assessment: summary information on overall survival, disease free or progression free survival with estimate of effect and 95% confidence interval.

### 2.5 Methodological Quality and Risk of Bias Assessment

Two independent investigators (AS and HE) evaluated the quality of the included meta-analysis using the Assessing the Methodological Quality of Systematic Reviews version 2.0 (AMSTAR 2.0) checklist. The AMSTAR 2.0 includes 16 items categorised into critical and non-critical domains allowing to rate studies based on weaknesses in critical domains. Confidence in the results of the studies is classified as high, moderate, low, or critically low confidence instead of using an overall score (27).

Risk of bias was also examined by means of the ‘Risk of Bias in Systematic Reviews’ (ROBIS) tool which is completed in three phases: (1) assess relevance (optional), (2) identify concerns with the review process, and (3) judge risk of bias. Our analysis comprised phase 2 and 3 which cover four domains through which bias may be introduced into the review, namely study eligibility criteria; identification and selection of studies; data collection and study appraisal; synthesis and findings as well as the overall risk of bias in the interpretation of review findings and whether priorly identified limitations were taken into consideration (28).

### 2.6 Statistical Analysis

We synthesised the data from the meta-analyses in terms of: search period, type of intervention, comparison, primary and secondary outcome, number of included randomised controlled trials and relative risk estimates including odds ratio (OR), and/or hazard ratio (HR).

For each meta-analysis, we synthesised the summary effect as well as the 95% confidence interval (CI). We quantified the degree of heterogeneity using the I2 statistic, which represents the percentage of the total variability across studies which is due to heterogeneity. I2 values of 25%, 50% and 75% corresponded to low, moderate and high degrees of heterogeneity respectively. Subgroup analysis was conducted on the basis of primary and recurrent ovarian cancer. Publication bias was assessed through examining asymmetry in the funnel plot. All statistical analyses were performed using Revman 5.3. Ethical approval was not necessary as this study did not involve patient consent.

## 3 Results

### 3.1Summary of Meta-Analyses

**Figure 1:**
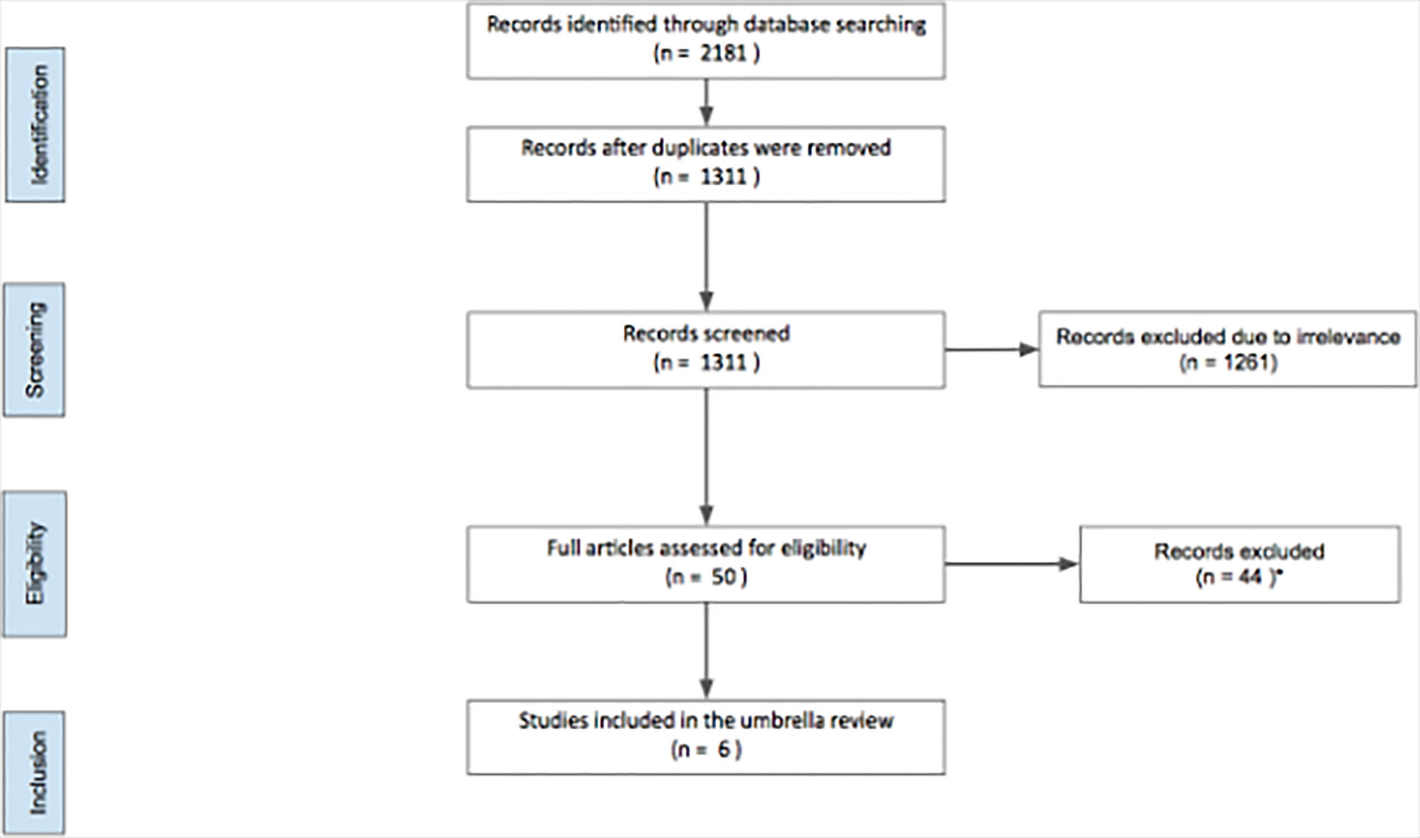
Search flowchart. *A list of excluded studies is provided in **Appendix 1** **(List of excluded articles Appendix)**.

We initially identified 1311 articles, of which 1261 were excluded as considered irrelevant to our search for not addressing CRS and HIPEC according to title and abstract screening. Among 50 articles examined in full text, 44 were not included in the umbrella review as they did not meet the inclusion criteria decided in the protocol (**List of excluded articles Appendix**). We ultimately identified 6 meta-analyses investigating the association of HIPEC with CRS in the management of ovarian cancers. Five meta-analyses reported overall survival outcomes. Moreover, 2 meta-analyses assessed disease free survival; 3 investigated progression free survival; and one studied both. **Figure 1** shows the process of study selection. The publication dates of eligible meta-analyses ranged from 2015 to 2020. As the methodological quality of the meta-analyses is relatively equal, we disregarded the overlap in some primary studies to avoid excluding eligible meta-analyses. The characteristics of the extracted data is presented in **Table 1**.

**Table 1:** Summary of selected meta-analyses.

Review (year)	assessed time periods	Intervention	Comparison	Number of studies	Number of RCTs	Outcomes	Hazard ratio(95% CI)	Heterogeneity I2 (P value)
Huo et al. (2015) (29)	2000 to 2015	Hyperthermic intraperitoneal chemotherapy combined to cytoreductive surgery	Cytoreductive surgery and chemotherapy	37	1 RCT	Primary: overall survival and disease free survival in general	**OS**: 2,53 (1,28-5) **DFS**: -	I2 = 46% (p = 0,1)
						Secondary: morbidity/mortality and quality of life.	–	–
						Subgroup analysis: primary or recurrent ovarian cancer Completeness of cytoreductive surgery, platinum resistance, chemotherapy agents,	–	–
Wang et al. (2019) (30)	2004 to 2018	Hyperthermic intraperitoneal chemotherapy	Cytoreductive surgery	76	2 RCTs	Primary: Overall survival and disease free survival in general and in primary or recurrent ovarian cancer separately.	**OS**: 0,56 (0,41-0,76) **DFS**: 0,61 (0,48-0,77)	I2 = 39% (p = 0,081) I2 = 26,3% (p = 0,210)
						Secondary: Adverse events, morbidity, mortality and quality of life.	–	–
						Subgroup analysis: Primary and recurrent ovarian cancer, initial stage, residual tumour, different HIPEC regimens (drugs, temperature, duration and timing).	Primary: **OS**: 0.57 (0.40, 0.83) **DFS**: 0.61 (0.47, 0.80) Recurrent: **OS**: 0.48 (0.24, 0.96) **DFS**: 0.59 (0.33, 1.08)	I2 = 49,5% (p = 0,04) I2 = 32,39% (p = 0,01) I2 = 43,5% (p = 0,01) I2 = 39,5% (p = 0,09)
Zhang et al. (2019) (31)	2004 to 2018	Hyperthermic intraperitoneal chemotherapy	Patients treated with traditional treatment without HIPEC	13	2 RCTs	Primary: overall survival and progression free survival.	**OS**: 0.54 (0.45 - 0.66) **PFS**: 0,45 (0,32-0,62)	I2 = 48% (p = 0,03) I2 = 60% (p = 0,02)
						Secondary: -	–	–
						Subgroup analysis: The effect of HIPEC on primary *vs* recurrent disease, stage III and IV cancer, the influence of CC3 and HIPEC timing.	Primary: **OS**: 0.59 (0.46 - 0.72) **PFS**: 0.41 (0,32-0,54) Recurrent: **OS**: 0.45 (0.24 - 0.83) **PFS**: 0,55 (0,27-1,1) Stage III-IV: **OS**: 0,64 (0,50-0,82) **PFS**: 0,36 (0,20-0,65) Interval CRS + HIPEC: **OS:** 0,61 (0,45-0,83) **PFS:** 0,29 (0,1-0,86) Primary CRS+HIPEC: **OS:** 0,47 (0,37-0,61) **PFS:** 0,52 (0,41-0,65) Without CC3: **OS:** 0,43 (0,33-0,55) **PFS:** 0,43 (0,33-0,55)	I2 = 34% (p <0.0001) I2 = 32% (p <0.0001) 60% (p = 0,01) 77% (p = 0,09) 0% (p = 0,004) 66% (p = 0,007) 50% (p = 0,002) 80% (p = 0,03) 50% (p<0,0001) 50% (p<0,0001) 28% (p<0,0001) 0% (p<0,0001)
Wu et al. (2019) (32)	2004 to 2018	Hyperthermic intraperitoneal chemotherapy	Patients with EOC treated without HIPEC	17	2 RCTs	Primary: overall survival and progression free survival.	**OS**: 0.50 (0.36 - 0.69) **PFS**: 0,57 (0,47-0,69)	I2 = 45,7% (p = 0,032) I2 = 21,3% (p = 0,247)
						Secondary: Yearly rate of survival.	–	–
						Subgroup analysis: Effect of HIPEC according to different study designs and primary or recurrent disease	Primary: **OS**: 0,59 (0,37-0,96) **PFS**: 0,57 (0,40-0,70) Recurrent: **OS**: 0,39 (0,24-0,65) **PFS**: 0,60 (0,39-0,91)	I2 = 53% (p = 0,075) I2 = 9,9% (p = 0,353) I2 = 44,2% (p = 0,084) I2 = 48,8% (p = 0,118)
Kim et al. (2019) (33)	2004 to 2018	Hyperthermic intraperitoneal chemotherapy	Patients treated without HIPEC	15	2 RCTs	Primary: overall survival and disease free survival.	**OS**: 0.640 (0.519–0.78) **DFS**: 0.603 (0.513–0.70)	I2 < 48,58 (p = 0,025) I2 <36 (p = 0,120)
						Secondary: -	–	–
						Subgroup analysis: effect of HIPEC for primary or recurrent disease and in case control studies	Primary: **OS**: 0,611 (0,376-0,992) **DFS**:0,580 (0,476-0,706) Recurrent: **OS**: 0,566 (0,379-0,844) **DFS**:0,644 (0,395-1,049) Case control studies: **OS**: 0,613 (0,396-0,944) **DFS**: 0,575 (0,471-0,702)	I2<63,56 (p = 0,027) I2<0,001 (p = 0,679) I2<38,91 (p = 0,132) - I2<55,52 (p = 0,013) -
Bouchard-Fortier et al. (2019) (34)	to 2019	Hyperthermic intraperitoneal chemotherapy	Patients with EOC treated without HIPEC	35	1 RCT	Primary: overall survival and progression free survival	**OS:** 3-year OS range: 46–77% **PFS:** 3-year PFS range: 17%–63%	
						Secondary: proportion of patients experiencing grade III-IV adverse events by 30 days postoperatively, proportion of patients who died by 30 days postoperatively and proportion of patients who had a reoperation within 30 days postoperatively	**30 days morbidity:** 34% (95% CI 20–52) **30 days Postoperative death:** 0% (95% CI 0–5) **30 days Reoperation rate:** 8% (95% CI 4–15)	I2 = 73 (p<0,01) I2 = 58 (p = 1) -

### 3.2 Methodological Quality of Included Reviews

Using the **AMSTAR 2** quality checklist, only 2 meta-analyses had a priorly registered protocol, and 3 studies had a comprehensive literature search strategy conducted in at least two bibliographic databases and supplemented by searching grey literature. None of the meta-analyses provided a list of excluded studies. Overall, 1 meta-analysis had low quality rating, while the 5 other meta-analyses had a critically low quality. Detailed findings from the AMSTAR 2 analysis are summarised in **Table 2**.

**Table 2:** Findings of the AMSTAR quality checklist.

AMSTAR QUESTIONS	Bouchard-Fortier et al. (34)	Wang et al. (30)	Zhang et al. (31)	Wu et al. (32)	Huo et al. (29)	Kim et al. (33)
**Q 1**	Yes	Yes	Yes	Yes	Yes	Yes
**Q 2**	Partial Yes	No	No	Yes	No	No
**Q 3**	No	No	No	No	No	No
**Q 4**	Partial Yes	No	No	Partial Yes	Partial Yes	No
**Q 5**	Yes	Yes	Yes	Yes	Yes	Yes
**Q 6**	Yes	Yes	Yes	Yes	Yes	Yes
**Q 7**	No	No	No	No	No	No
**Q 8**	Yes	Yes	Yes	Yes	Yes	Yes
**Q 9**	Yes	No	Yes	Yes	Yes	No
	Yes	Yes	Yes	Yes	–	Yes
**Q 10**	No	No	No	No	No	No
**Q 11**	Yes	Yes	Yes	Yes	Yes	Yes
	N/A	Yes	Yes	Yes	–	Yes
**Q 12**	No	No	Yes	Yes	Yes	No
**Q 13**	No	No	Yes	Yes	Yes	Yes
**Q 14**	Yes	Yes	Yes	Yes	Yes	Yes
**Q 15**	No	Yes	Yes	Yes	Yes	Yes
**Q 16**	Yes	Yes	Yes	Yes	Yes	Yes
**Critical**	3	4	3	1	2	3
**Non critical**	3	3	2	2	2	3
**Level**	Critically low	Critically low	Critically low	low	Critically low	Critically low

### 3.3 Risk of Bias Assessment

We assessed the risk of bias in all included meta-analyses and all 6 studies had low risk of bias. Details are shown in **Table 3**.

**Table 3:** Risk of bias assessment.

ROBIS DOMAINS	Bouchard-Fortier et al. (34)	Huo et al. (29)	Wang et al. (30)	Zhang et al. (31)	Wu et al. (32)	Kim et al. (33)
DOMAIN 1: STUDY ELIGIBILITY CRITERIA						
1.1 Did the review adhere to pre-defined objectives and eligibility criteria?	Yes	Probably yes	Probably yes	Probably yes	Yes	Probably yes
1.2 Were the eligibility criteria appropriate for the review question?	Yes	Yes	Yes	Yes	Yes	Yes
1.3 Were eligibility criteria unambiguous?	Yes	Yes	Yes	Yes	Yes	Yes
1.4 Were any restrictions in eligibility criteria based on study characteristics appropriate (e.g. date, sample size, study quality, outcomes measured)?	Yes	Yes	Yes	Yes	Yes	Yes
1.5 Were any restrictions in eligibility criteria based on sources of information appropriate (e.g. publication status or format, language, availability of data)?	Yes	Yes	Probably yes	Probably yes	Yes	Probably yes
Concerns regarding specification of study eligibility criteria	Low concern	Low concern	Low concern	Low concern	Low concern	Low concern
DOMAIN 2: IDENTIFICATION AND SELECTION OF STUDIES						
2.1 Did the search include an appropriate range of databases/electronic sources for published and unpublished reports?	Yes	Yes	Yes	Yes	Yes	Yes
2.2 Were methods additional to database searching used to identify relevant reports?	Yes	Yes	Yes	Yes	Yes	No information
2.3 Were the terms and structure of the search strategy likely to retrieve as many eligible studies as possible?	Yes	Probably no	Probably no	Probably yes	Probably no	Probably yes
2.4 Were restrictions based on date, publication format, or language appropriate?	Probably yes	Probably yes	no	no	Probably yes	no
2.5 Were efforts made to minimise error in selection of studies?	Yes	Yes	no	Yes	Yes	Yes
Concerns regarding specification of study eligibility criteria	Low	Low	High	Low	Low	Low
DOMAIN 3: DATA COLLECTION AND STUDY APPRAISAL						
3.1 Were efforts made to minimise error in data collection?	Yes	Yes	Yes	Yes	Yes	Yes
3.2 Were sufficient study characteristics available for both review authors and readers to be able to interpret the results?	Yes	Yes	Yes	Probably yes	Probably yes	Probably no
3.3 Were all relevant study results collected for use in the synthesis?	Yes	Yes	Yes	Yes	Yes	Yes
3.4 Was risk of bias (or methodological quality) formally assessed using appropriate criteria?	Yes	Probably yes	Yes	Probably yes	Probably yes	Probably yes
3.5 Were efforts made to minimise error in risk of bias assessment?	Yes	Probably no	Probably no	Probably no	Probably no	Probably no
Concerns regarding specification of study eligibility criteria	Low	Low	Low	Low	Low	Low
DOMAIN 4: SYNTHESIS AND FINDINGS						
4.1 Did the synthesis include all studies that it should?	Probably yes	Yes	Yes	No information	Probably yes	Probably yes
4.2 Were all pre-defined analyses reported or departures explained?	Yes	No	No	No	Yes	No
4.3 Was the synthesis appropriate given the nature and similarity in the research questions, study designs and outcomes across included studies?	Yes	Yes	Yes	Yes	Yes	Yes
4.4 Was between-study variation (heterogeneity) minimal or addressed in the synthesis?	Yes	Yes	Yes	Yes	Yes	no
4.5 Were the findings robust, e.g. as demonstrated through funnel plot or sensitivity analyses?	Yes	Yes	Yes	Yes	Yes	Yes
4.6 Were biases in primary studies minimal or addressed in the synthesis?	Probably not	No	No	No	No	No
Concerns regarding specification of study eligibility criteria	Low	Low	Low	Low	Low	High
RISK OF BIAS IN THE REVIEW						
A. Did the interpretation of findings address all of the concerns identified in Domains 1 to 4?	Yes	Yes	Probably yes	Yes	Yes	Probably yes
B. Was the relevance of identified studies to the review’s research question appropriately considered?	Yes	Yes	Yes	Yes	Yes	Yes
C. Did the reviewers avoid emphasizing results on the basis of their statistical significance?	Probably yes	Probably yes	Probably yes	Probably yes	Probably yes	Probably yes
Overall risk of bias in the review	Low	Low	Low	Low	Low	Low

### 3.4 Significant Findings and Heterogeneity

#### 3.4.1 Overall Survival

**Figure 2:**
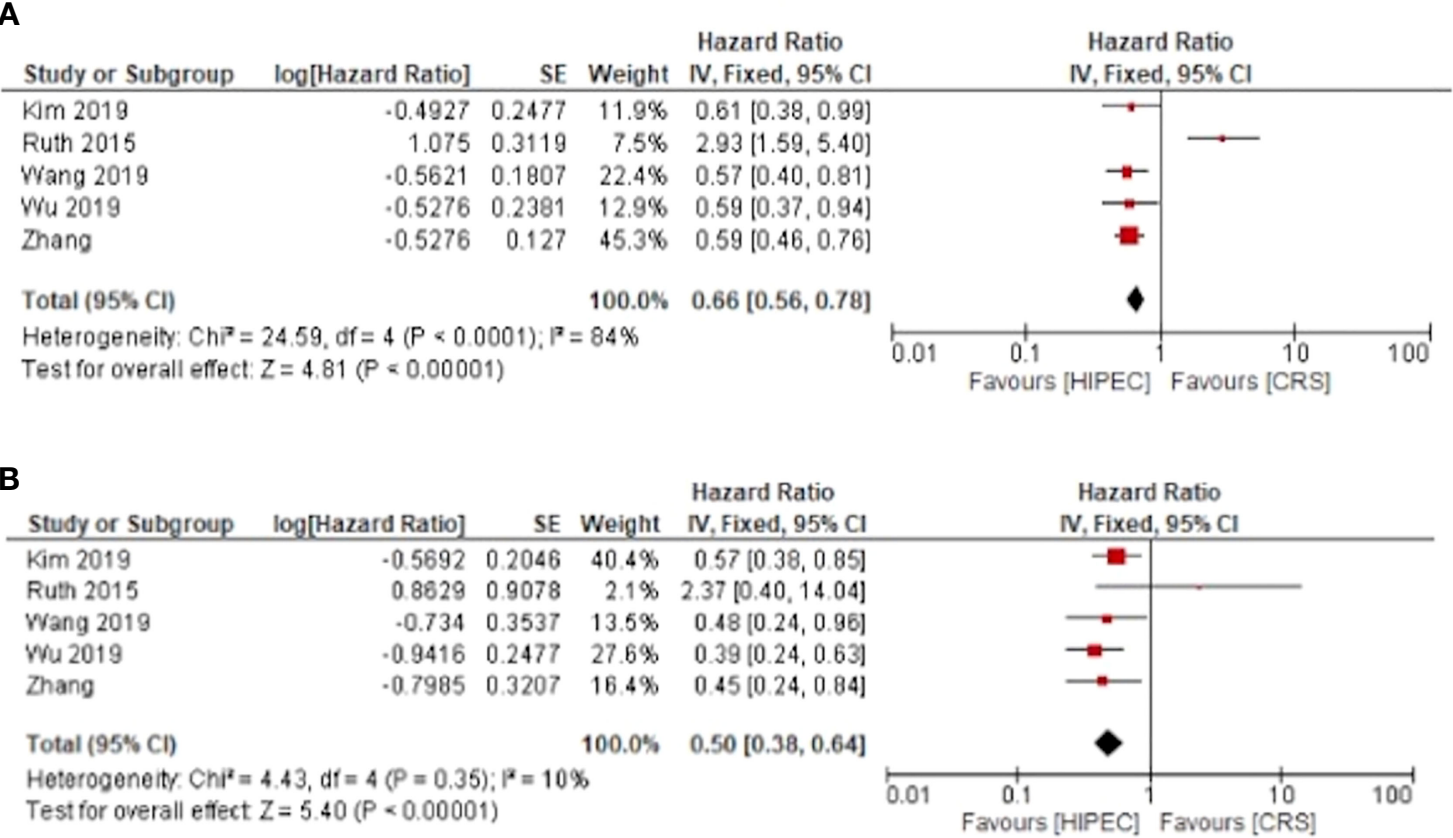
HIPEC + CRS versus CRS overall survival forest plots **(A)** primary ovarian cancer **(B)** recurrent ovarian cancer.

Five of the included studies investigated the overall survival of patients undergoing HIPEC + CRS in comparison to CRS alone, with separated outcomes for primary and recurrent ovarian cancer. The meta-analyses showed that HIPEC + CRS was associated with improved 3 year survival for primary and recurrent cancers with calculated pooled hazard ratios and 95% confidence intervals of HR: 0.66, 95%CI: 0.56-0.78 and HR: 0.50, 95%CI: 0.38-0.64 respectively (**Figure 2**).

#### 3.4.2 Disease Free Survival

**Figure 3:**
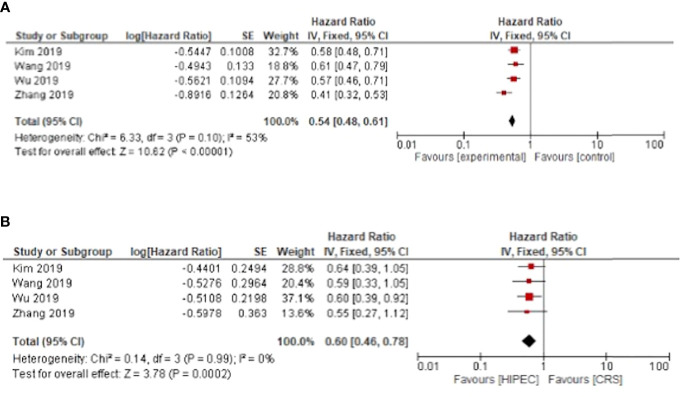
HIPEC + CRS versus CRS disease free survival forest plots **(A)** primary ovarian cancer **(B)** recurrent ovarian cancer.

Only four meta-analyses investigated disease free survival. HIPEC + CRS was associated with a disease-free survival benefit for primary and recurrent ovarian cancer with pooled hazard ratios of HR: 0.54, 95%CI: 0.48-0.61 and HR: 0.60, 95%CI: 0.46-0.78 respectively (**Figure 3**).

#### 3.4.3 Progression Free Survival

**Figure 4:**
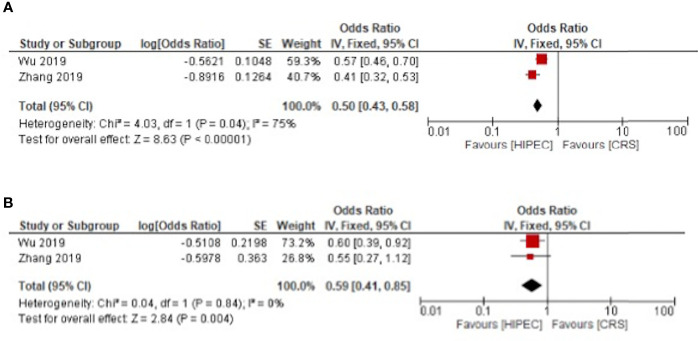
HIPEC + CRS versus CRS progression free survival forest plots **(A)** primary ovarian cancer **(B)** recurrent ovarian cancer.

The progression free survival was improved for primary and recurrent ovarian cancer respectively with HR: 0.50, 95%CI: 0.43-0.58 and HR: 0.59, 95%CI: 0.41-0.85 (**Figure 4**).

#### 3.4.4 Heterogeneity Assessment

For primary ovarian cancer, heterogeneity was present for both overall and disease-free survival at three years with I²= 84%, p < 0.01 and I²= 53%, p = 0.1 respectively. Regarding recurrent cancers, the heterogeneity was low for overall survival with I²= 10%, p = 0.35. Heterogeneity was not present for disease free and progression free survival in recurrent cancer (**Figures 3** and **4**).

#### 3.4.5 Morbidity

Y. Wang et al. qualitatively reviewed available data on adverse events and morbidity in patients undergoing HIPEC and CRS. On the other hand, the meta-analyses by G.Bouchard Fortier et al. and Y.R. Huo et al. calculated in primary EOC settings, the pooled proportions of 30-day grade III-IV morbidity, estimated at 34% (95% CI 20-52) and 31.3% (range: 1.8-55.6%) respectively. In addition, Y.R. Huo et al. reported pooled Grade III-IV morbidity rate of 26.2% (1.8-55.6%) in recurrent settings.

#### 3.4.6 Quality of Life

Both the meta-analyses by Y.Ruth and Y. Wang reported studies assessing quality of life in patients undergoing HIPEC + CRS with the following quality of life assessment tools developed by the European Organization for Research and Treatments of Cancer (EORTC): Quality of Life Questionnaire-Core 30 (QLQ-C30), Quality of Life Questionnaire-OVarian Cancer Module (QLQ-OV28), Quality of Life Questionnaire-ColoRectal Cancer Module (QLQ- CR38) and the V FACT-QOL questionnaire. Overall, although data on quality of life remains insufficient, patients undergoing HIPEC + CRS either showed no significant difference, or a worsening followed by an improvement within 90% of the baseline by 6 months but while remaining below the baseline (p<0.05).

## 4 Discussion

The addition of HIPEC to CRS has been discussed as a potential therapeutic option for peritoneal malignancy management (12, 16, 35–37). The efficacy of HIPEC would result from the specific ovarian cancer’s pattern of spread, the heat effect on anticancer drugs permeability, and the increased chemosensitivity of cancer cells to heat therapeutics (38, 39). This is the first umbrella review of meta-analyses giving an overview and synthesis of the available evidence on the association of HIPEC and CRS in the management of primary and recurrent ovarian cancer.

Our umbrella review confirms the benefit of using CRS and HIPEC for the management of primary ovarian cancer on overall survival, disease free and progression free survival. These results are in line with the OVHIPEC 1 study outcomes by Van Driel et al. (16). The use of HIPEC was associated with a 25% reduction in relapse (HR: umbrella 0.54 vs Van Driel 0.68) and death risk (HR: umbrella 0.66 *vs* Van Driel 0.67). Moreover, although the relative effect of HIPEC is more marked on recurrence-free survival compared to overall survival, the absolute benefit is higher on overall survival. Despite the existence of high-quality evidence supporting the use of HIPEC, numerous questions are still pending, such the optimal surgery timing between immediate or interval surgery when HIPEC is to be combined to CRS, along with the best time-point for CRS and HIPEC, or the optimised doses and temperatures to be used.

Regarding recurrent ovarian cancers, the HIPEC + CRS association was also associated with overall survival, disease free and progression free survival improvements compared to CRS alone. In fact, the utility of HIPEC for survival has previously been reported by Spiliotis and al (40) in patients with platinum-sensitive, or platinum-resistant recurrent ovarian cancers. Nonetheless, these results were criticised because the statistical hypothesis and primary endpoints had not been clearly defined (41), thereby urging the need for higher scientific evidence, which should be provided by the results from the Italian trial HORSE (42) and the French randomised trial CHIPOR (43)

Historically, HIPEC was developed as an alternative to the increasing use of intraperitoneal chemotherapy (IP) (44) in the 2010’s, which explains the frequent comparison of their mechanisms. That being said, the efficacy of the IP chemotherapy has been reconsidered following the GOG 252 study (45). The randomised trial comparing Intravenous Versus IP Chemotherapy in addition to Bevacizumab showed no disease free survival (DFS) improvement with IP therapy, irrespective of the residual tumour size following CRS. The DFS of patients receiving intravenous chemotherapy, IP Carboplatin and IP Cisplatin were 31.3 months, 31,8 and 33,8 months respectively, therefore showing no significant benefit. A major difference between HIPEC and IP chemotherapy relates to the repetitive administration of chemotherapy in the peritoneum through an intraperitoneal catheter, in addition to the IC administration. Platinum salts, especially cisplatin, are characterised by a strong absorption from peritoneum to blood which limits the peritoneal lesion drug exposure while inducing strong systemic side effects, eventually interfering with the dose intensity of the systemic chemotherapy. Moreover, the peritoneum catheter is associated with local complications such as numerous adhesions formation, infection and pain that hampers extensive locoregional chemotherapy. In contrast, HIPEC is a targeted locoregional therapy meant to complement radical surgery and allow an exhaustive treatment for peritoneal surface malignancies at the end of surgery. Indeed, the surgical procedure is most likely associated with cancer cells’ release within the peritoneal cavity, which could be effectively eliminated by the local application of chemotherapy after the procedure. This may explain why the survival improvement observed with HIPEC may not be the result of an additional Cisplatin dose (46). The systemic passage handoff HIPEC is limited by the decreased duration of exposure, as the abdomen is washed out at the end of the procedure, in addition to the use of Thiosulfate to mitigate Cisplatin-induced toxicity, the reason why this type of treatment is now recommended.

Wang et al’s review on morbidity in patients undergoing HIPEC and CRS, along with two studies, namely Cascales-Campos et al. (47) and Munoz-Casares (48), agreed on the similarity of the overall postoperative morbidity rate between patients undergoing HIPEC and CRS. On the other hand, a study by Ryu et al. reported a non-statistically significant increased rate of major complications for patients with HIPEC (49), while another study reported more grade III-IV complications in the HIPEC group (P=0.02) (50). These complications included minor leaks, ileus, transient hepatitis, leucopenia, abdominal pain, infection and fistulas (30, 32).

Regarding the complications of HIPEC procedures, only 2 meta-analyses reported the 30 days rate of complications for grade III and IV with Y.R Huo et al. (29) describing separate morbidity pooled rates for primary and recurrent EOC of 31.3% (range: 1.8-55.6%) and 26.2% (1.8-55.6%), while G. Bouchard Fortier et al. (34) rounded up the 30 day morbidity pooled rate for primary and recurrent epithelial cancer to 34% (95% CI 20–52). These complication rates are thought to be more related to the effects of extensive CRS rather than the HIPEC therapy (34), especially as Van Driel was the only study comparing the adverse events rates for patients undergoing surgery alone and those with combined surgery and HIPEC and where no statistically significant difference was noted (25% *vs.* 27%, p = 0.76).

Both the meta-analyses by Y. Ruth (29) and Y. Wang (30) described the quality of life in patients undergoing HIPEC + CRS. Using the V FACT-O QoL questionnaire, Huo et al. showed that if the immediate post-operative quality of life following surgery was worse than the presurgery period (FACT-O score 126 *vs* 108, p<0.05), it improved later with a 90% baseline improvement at 6 months post-surgery (p<0.05) (29, 51). The European Organization for Research and Treatments of Cancer (EORTC) Quality of Life Questionnaire-Core 30 (QLQ-C30), Quality of Life Questionnaire-OVarian Cancer Module (QLQ-OV28) and Quality of Life Questionnaire-ColoRectal Cancer Module (QLQ-CR38) were also used to assess quality of life following HIPEC. No visible quality of life difference between HIPEC + CRS and CRS only patients were observed (16). In another study, Chia et al. used the EORTC QLQ-C3o to assess the quality of life of HIPEC + CRS patients and demonstrated a decrease in quality of life, particularly of the physical and role functioning scales (52). However, this decline was short in time, and improvement or return to baseline 6-12 months after surgery was observed in most cases (52–54). Risk factors associated with worse Qol were higher age, prolonged operation time, extensive disease, residual disease, adjuvant chemotherapy, complications, stoma placement, and recurrent disease (53).

The present study has some limitations. The main weakness linked to HIPEC studies resides in the heterogeneity of the used intraperitoneal regimens, in terms of chemotherapy, dose, temperature and timing with relation to neoadjuvant treatment. For example, while a phase 1 dose-escalation found that cisplatin optimal dose was 70 mg/m2 (55), a subsequent phase II reported 40% morbidity rate using cisplatin at 75 mg/m2 (56). These discrepancies make the data difficult to interpret when we have to choose the best regimen, especially as the difference in applied HIPEC regimens by each institute may be one of the reasons for different treatment outcomes. That being said, several networks are carrying out the work of harmonising these protocols into more standardised recommendations. Some additional analyses would have been interesting in the present project, such as the assessment of BRCA status distribution and the comparison of HIPEC regimens, CRS-HIPEC intervals and whether CRS was primary debulking or interval debulking. However, they were not possible since these data were not examined in the included studies. It would also be useful to develop criteria for the selection of patients, surgeon’s level of experience, as well as the choice of cytostatic (such as dose, temperature, duration of cytostatic application) in order to standardise regimens. Another important limitation relates to the current change of practice with the increasing prescription of PARP inhibitors both in first line and recurrent settings. None of the studies we investigated assessed the role of PARP inhibitors in patients treated with CRS and HIPEC. Well designed RCT are warranted to confirm that CRS + HIPEC and PARP inhibitors can be safely combined, and that HIPEC still contributes to improving PFS and OS. Another limitation is the reduced number of meta-analyses and RCTs which were published in a close interval of time, nevertheless, the umbrella review was conducted with the aim of contrasting the results from these studies as determined in the forest plots. The umbrella review methodology that we used also integrates limitations. The meta‐analyses in this umbrella review contained a small proportion of randomised controlled trials compared to the number of observational studies, which could have decreased the quality of evidence. The methodological quality of the included meta-analyses is overall considered to be low, thereby urging research teams to improve the methodological quality of future meta-analyses by following methodology guidelines, especially when addressing controversial subjects.

Despite these limitations, the implementation of HIPEC in routine is facing an opposition exerted by many clinicians beyond scientific rationality and objectivity. A part of the problem may be related to the natural reluctance of teams to change their organisations, along with conflicts between gynaecologic oncologists and general surgeons. The latter ones Indeed initiated the HIPEC technology for GI cancers with peritoneal involvement before subsequently developing this technique for gynaecologic cancers with their experience of peritoneal carcinomatosis management. This evolution might have led to some conflicts with gynaecologists. In that context, further evidence confirming or not the benefit related to HIPEC will be needed to help the ovarian cancer specialists agree on the utility of this approach.

At present, the utility of heated intraperitoneal chemotherapy in the management of epithelial ovarian cancer (EOC) is still a controversial topic in the scientific community, which pending prospective studies are expected to settle. However, for the time being, the present umbrella study suggests that HIPEC performed at the end of CRS seems to benefit patients treated for primary disease after neoadjuvant chemotherapy as demonstrated in OVHIPEC-1. Further studies are awaited to assess HIPEC in recurrent settings and confirm its utility in primary ovarian cancer.

## Data Availability Statement

The original contributions presented in the study are included in the article/**Supplementary Material**. Further inquiries can be directed to the corresponding author.

## Author Contributions

Conceptualization: AS, HE, and NB. Data curation: AS and HE. Formal analysis, AS, HE, and MM. Investigation: AS and HE. Methodology: AS, HE, and MM. Project administration: AS. Supervision: HE and NB. Validation: AB, SB, BY, OG, and RM. Writing—original draft: AS, HE, and NB. Writing—review and editing: MM, AB, SB, BY, OG, and RM. All authors contributed to the article and approved the submitted version.

## Funding

Publication fees were supported by the ARCO (association pour la recherche et l'enseignement en chirurgie oncologique) and ThermaSolutions.

## Conflict of Interest

The authors declare that the research was conducted in the absence of any commercial or financial relationships that could be construed as a potential conflict of interest.

## Publisher’s Note

All claims expressed in this article are solely those of the authors and do not necessarily represent those of their affiliated organizations, or those of the publisher, the editors and the reviewers. Any product that may be evaluated in this article, or claim that may be made by its manufacturer, is not guaranteed or endorsed by the publisher.
