# Supplementary material for: Hyperthermic Intraperitoneal Chemotherapy and Cytoreductive Surgery in Ovarian Cancer: An Umbrella Review of Meta-Analyses
Source: Front Oncol. 2022 May 9;12:809773. doi: 10.3389/fonc.2022.809773 (PMC9124965; doi:10.3389/fonc.2022.809773)
Supplement: Supplementary file 1 [file DataSheet_1.docx]

# Supplementary Materials :

Appendix 1 : search strategy and list of excluded studies

## Search strategy :

Search strategy for MEDLINE :

#1: "Ovarian Neoplasms"

#2: “Ovary”

#3: “"Carcinoma, Ovarian Epithelial”

#4: #1 OR #2 OR #3

#5: "Hyperthermia, Induced"

#6: "Meta-Analysis"

#7: "Meta-Analysis as Topic"

#8 : #6 OR #7

#9 : #4 AND #5 AND #8

Results : 18894 articles found

With meta-analysis filter : 1869 articles found

Search strategy for the Cochrane Library :

#1: "Ovarian Neoplasms"

#2: “Ovarian cancer”

#3: #1 OR #2

#4: "Hyperthermia, Induced"

#5: “HIPEC”

#6: #4 OR #5

#7: "Meta-Analysis"

#8: #3 AND #6 AND #7

Results : 51 cochrane reviews

Search strategy for Scopus :

#1: "Ovarian Neoplasms"

#2: “Ovarian cancer”

#3: #1 OR #2

#4: “HIPEC”

#5: "Hyperthermia, Induced"

#6: #4 OR #5

#7: “Meta-analysis”

#8: #3 AND #6 AND #7

Results : 17 articles found

Search strategy for Web of science :

#1: "Ovarian Neoplasms"

#2: “Ovarian cancer”

#3: #1 OR #2

#4: “HIPEC”

#5: "Hyperthermia, Induced"

#6: #4 OR #5

#7: “Meta-analysis”

#8: #3 AND #6 AND #7

Results : 13 articles found

Search strategy for Science direct:

#1: "Ovarian Neoplasms"

#2: “Ovarian cancer”

#3: “HIPEC”

#4: "Hyperthermia, Induced"

#5: “ meta-analysis”

#6: #2 AND #3 AND #5

Results : 161 articles found

#7: #1 AND #3 AND #5

Results : 56 articles found

Search strategy for Prospero :

#1: ovarian cancer

#2: ovarian neoplasm

#3: #1 OR #2

#4: HIPEC

#5: Hyperthermic intraperitoneal chemotherapy

#5: #3 AND #4

Results : 8 protocoles

#6: #3 AND #5

Results : 5 protocoles

| References of excluded studies |
| --- |
| Picard et al., « CHIP et cancers de l’ovaire : pour quelles patientes ? » |
| Fagotti et al., « Secondary cytoreduction plus oxaliplatin-based HIPEC in platinum-sensitive recurrent ovarian cancer patients: A pilot study ». |
| Bakrin et Gladieff, « Tumeurs épithéliales malignes de l’ovaire : place de la chimiothérapie intrapéritonéale et de la chimiothérapie hyperthermique intrapéritonéale (CHIP). Article rédigé sur la base de la recommandation nationale de bonnes pratiques cliniques en cancérologie intitulée « Conduites à tenir initiales devant des patientes atteintes d’un cancer épithélial de l’ovaire » élaborée par FRANCOGYN, CNGOF, SFOG, GINECO-ARCAGY sous l’égide du CNGOF et labellisée par l’INCa ». |
| Chua et al., « Establishing evidence for change in ovarian cancer surgery — Proposing clinical trials of cytoreductive surgery and hyperthermic intraperitoneal chemotherapy (HIPEC) in ovarian cancer peritoneal carcinomatosis » |
| Petrillo et al., « Secondary cytoreductive surgery in patients with isolated platinum-resistant recurrent ovarian cancer: A retrospective analysis » |
| Piovano et al., « O546 CYTOREDUCTIVE SURGERY PLUS HIPEC IN PLATINUM-SENSITIVE RECURRENT OVARIAN CANCER PATIENTS: A CASE-CONTROL STUDY ON SURVIVAL IN PATIENTS WITH A TWO YEARS FOLLOW-UP » |
| Fagotti et al., « HIPEC in recurrent ovarian cancer patients: Morbidity-related treatment and long-term analysis of clinical outcome » |
| Fotopoulou et al., « HIPEC: HOPE or HYPE in the fight against advanced ovarian cancer? » |
| Picard et al., « CHIP et cancers de l’ovaire : pour quelles patientes ? », 2019 |
| Bakrin et al., « Hyperthermic intraperitoneal chemotherapy (HIPEC) in ovarian cancer » |
| Lavoue et al., « Management of epithelial cancer of the ovary, fallopian tube, primary peritoneum. Long text of the joint French clinical practice guidelines issued by FRANCOGYN, CNGOF, SFOG, GINECO-ARCAGY, endorsed by INCa. (Part 2: systemic, intraperitoneal treatment, elderly patients, fertility preservation, follow-up) » |
| Alyami et al., « Pressurised intraperitoneal aerosol chemotherapy: rationale, evidence, and potential indications » |
| Bakrin et al., « La chimiohyperthermie intrapéritonéale (CHIP) dans les cancers ovariens » |
| Bakrin et al., « Peritoneal carcinomatosis treated with cytoreductive surgery and Hyperthermic Intraperitoneal Chemotherapy (HIPEC) for advanced ovarian carcinoma: A French multicentre retrospective cohort study of 566 patients » |
| Sun et al., « Cytoreductive Surgery plus Hyperthermic Intraperitoneal Chemotherapy to Treat Advanced/Recurrent Epithelial Ovarian Cancer: Results from a Retrospective Study on Prospectively Established Database » |
| Gallotta et al., « Secondary Laparoscopic Cytoreduction in Recurrent Ovarian Cancer: A Large, Single-Institution Experience » |
| Carrabin et al., « Hyperthermic intraperitoneal chemotherapy with oxaliplatin and without adjuvant chemotherapy in stage IIIC ovarian cancer » |
| Bayon et al., « Cytoreductive surgery and hyperthermic intraperitoneal chemotherapy for the treatment of advanced epithelial ovarian carcinoma: Upfront therapy, at first recurrence, or later? » |
| Deraco et al., « Cytoreductive surgery and hyperthermic intraperitoneal chemotherapy as upfront therapy for advanced epithelial ovarian cancer: Multi-institutional phase-II trial » |
| Fagotti et al., « Role of cytoreductive surgery in recurrent ovarian cancer » |
| Ceelen et al., « Hyperthermic intraperitoneal chemoperfusion in the treatment of locally advanced intra-abdominal cancer » |
| Faluyi et Dickinson, « Interventions for the treatment of borderline ovarian tumours » |
| Coleridge et Morrison, « Chemotherapy versus surgery for initial treatment in advanced ovarian epithelial cancer » |
| Jaaback et Lawrie, « Intraperitoneal chemotherapy for the initial management of primary epithelial ovarian cancer » |
| Tangjitgamol et Bryant, « Interval debulking surgery for advanced epithelial ovarian cancer » |
| Galaal et Dickinson, « Cytoreductive surgery plus chemotherapy versus chemotherapy alone for recurrent epithelial ovarian cancer » |
| Chen et Feng, « Maintenance chemotherapy for ovarian cancer ». |
| Cistaro et al., « 18F-FDG PET/CT, Cytoreductive Surgery and Intraperitoneal Chemohyperthermia for the Therapeutic Management in Peritoneal Carcinomatosis ». |
| Horvath et al., « Cytoreductive Surgery and Hyperthermic Intraperitoneal Chemotherapy Combined with Liver Resection for Concurrent Peritoneal and Hepatic Metastases of Gastrointestinal and Gynecological Primary Tumors ». |
| Bristow, Puri, et Chi, « Cytoreductive Surgery for Recurrent Ovarian Cancer ». |
| Sun et al., « Cytoreductive Surgery plus Hyperthermic Intraperitoneal Chemotherapy to Treat Advanced/Recurrent Epithelial Ovarian Cancer ». |
| Dunn, « Cytoreductive Surgery With Hyperthermic Intraperitoneal Chemotherapy, Part I ». |
| Dellinger et al., « HIPEC Treatment in Advanced and Recurrent Ovarian Cancer ». |
| Almerey et al., « Intraoperative Fluid Restriction in Hyperthermic Intraperitoneal Chemotherapy ». |
| Jaaback et Johnson, « Intraperitoneal Chemotherapy for the Initial Management of Primary Epithelial Ovarian Cancer ». |
| Gadducci et Conte, « Intraperitoneal Chemotherapy in the Management of Patients with Advanced Epithelial Ovarian Cancer ». |
| Lowe et Morgan, « Intraperitoneal Chemotherapy of Ovarian Cancer ». |
| Ye et al., « Nephrotoxicity and Long-Term Survival Investigations for Patients with Peritoneal Carcinomatosis Using Hyperthermic Intraperitoneal Chemotherapy with Cisplatin ». |
| Pariury et al., « Pediatric Ovarian Angiosarcoma Treated with Systemic Chemotherapy and Cytoreductive Surgery with Heated Intraperitoneal Chemotherapy ». |
| Fujiwara et al., « Principle and Evolving Role of Intraperitoneal Chemotherapy in Ovarian Cancer ». |
| Zeimet et al., « Pros and Cons of Intraperitoneal Chemotherapy in the Treatment of Epithelial Ovarian Cancer ». |
| Gouy et al., « Results of a Multicenter Phase I Dose-Finding Trial of Hyperthermic Intraperitoneal Cisplatin after Neoadjuvant Chemotherapy and Complete Cytoreductive Surgery and Followed by Maintenance Bevacizumab in Initially Unresectable Ovarian Cancer ». |
| Marchetti et al., « Role of Intraperitoneal Chemotherapy in Ovarian Cancer in the Platinum-Taxane-Based Era ». |
